# Kaposi sarcoma (KS) with primary effusion lymphoma in HIV infected MSM (men having sex with men) co-infected with pulmonary tuberculosis and syphilis: a case report from India

**DOI:** 10.1186/s12981-022-00460-5

**Published:** 2022-07-16

**Authors:** Sampada Bangar, Rohit Vashisht, Pratiksha Sonar, Kunal Ghule, Leena Rawat, Arati Mane, Abhijit Kadam, Nomita Chandhiok, Seema Sahay

**Affiliations:** 1 ICMR- National AIDS Research Institute, Pune, India; 2 Armed Forces Medical College, Pune, India; 3 ART Center, Yashwantrao Chavan Memorial Hospital, Pune, India; 4 ART Center, Armed Forced Medical College, Pune, India; 5 Indian Council of Medical Research, Delhi, India

**Keywords:** MSM, Kaposi sarcoma, Primary effusion lymphoma (PEL), India, HAART

## Abstract

We describe a case of a 30-year-old MSM recently diagnosed with HIV, immunocompromised with a purplish or brown rash all over the body for 3 to 4 months. The histopathology of the cutaneous lesions and pleural effusion aspirate confirmed the diagnosis of Kaposi’s sarcoma (KS) and primary effusion lymphoma (PEL). While KS is one of the AIDS-defining illnesses seen in immunocompromised patients having low CD4 count, PEL is a rare and distinct subset of AIDS-related lymphoma. Despite the widespread availability of HIV testing, HIV diagnosis gets delayed due to stigma among MSM. This case report emphasizes the importance of early suspicion for symptoms of HIV-associated opportunistic infections in high-risk populations like MSM. The report reiterates the need for an ambient stigma-free environment for improving HIV screening in this high-risk population.

## Introduction

Kaposi’s sarcoma is a form of cancer that is commonly seen among patients with stage-4 human immunodeficiency virus (HIV) infection known as Acquired Immunodeficiency Syndrome (AIDS) [1]. The multicentric angioproliferative cancer of endothelial origin is associated with Human Herpesvirus 8 (HHV-8) infection and due to this association HHV-8 is also known as Kaposi sarcoma-associated herpesvirus 8 (KSHV8). The cancer is classified into four clinical types: Classic (Mediterranean), African (endemic), acquired immunodeficiency syndrome (AIDS)-associated (epidemic), and iatrogenic (transplant-related) [2, 3]. Classification of KS is based on the different populations it develops in, but the changes within the KS cells are very similar. The classical signs and symptoms include fever, joint pain, splenomegaly, lymphadenopathy, diarrhea, and fatigue. It was the first malignancy to be linked with Acquired Immunodeficiency Syndrome (AIDS). It was first diagnosed among HIV infected MSM population. In the Pre-Highly Active Antiretroviral Therapy (HAART) era, the prevalence of opportunistic infections was high among HIV-infected patients. With the advent of early initiation of HAART, a marked reduction in the incidence of KS is seen in HIV infected population however, an increasing trend in the incidence of KS is seen in high-risk populations like MSM [4]. Hence, a high level of suspicion is important for early diagnosis of KS in this high-risk population and timely linkage to care. In advanced cases, cancer may metastasize to the internal organs, due to immunosuppression. Primary effusion lymphoma (PEL) was first described in 1989 as an AIDS-related lymphoma. This AIDS-related lymphoma was also associated with HHV-8. PEL is an extra nodal non-Hodgkin’s lymphoma usually classified as a B-cell lymphoma that grows in the liquid phase within body cavities. It occurs mostly in immunocompromised patients, such as HIV-infected individuals and patients receiving organ transplantation. It belongs to the group of AIDS-related non-Hodgkin's lymphomas. The prognosis is poor and death often occurs within months of diagnosis.

## Case report

**Fig. 1:**
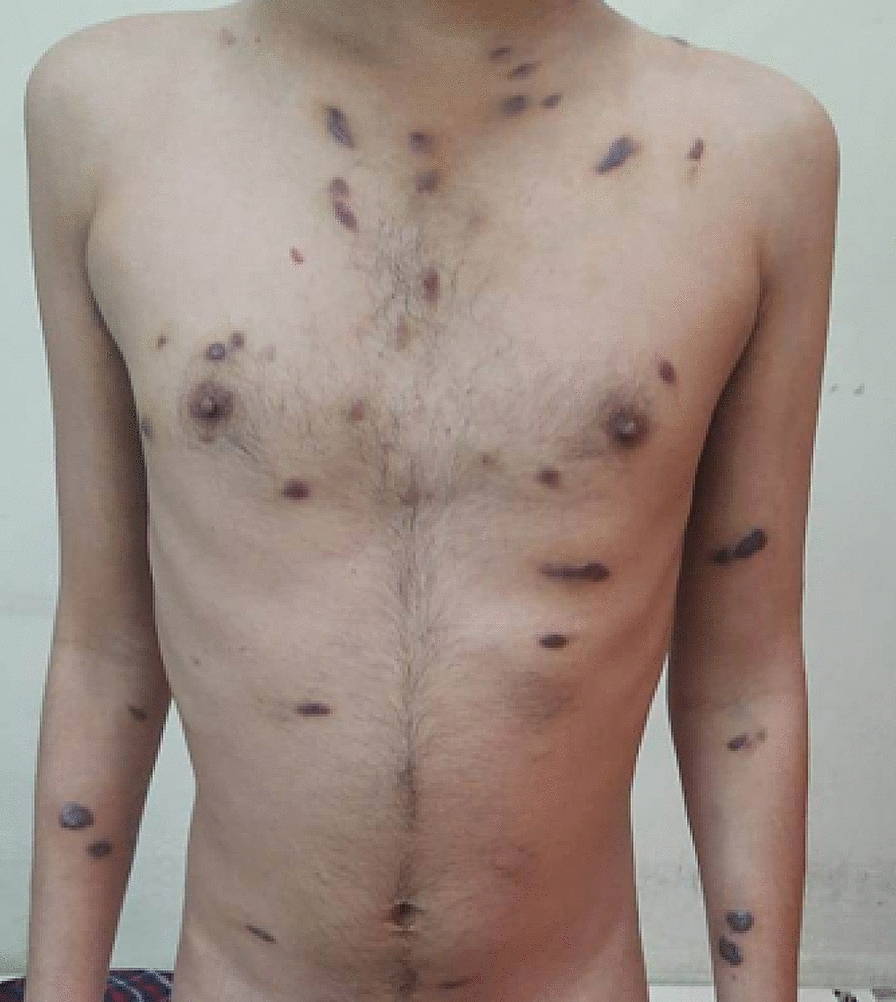
Purple, reddish-brown patches on the front side of the body

**Fig. 2:**
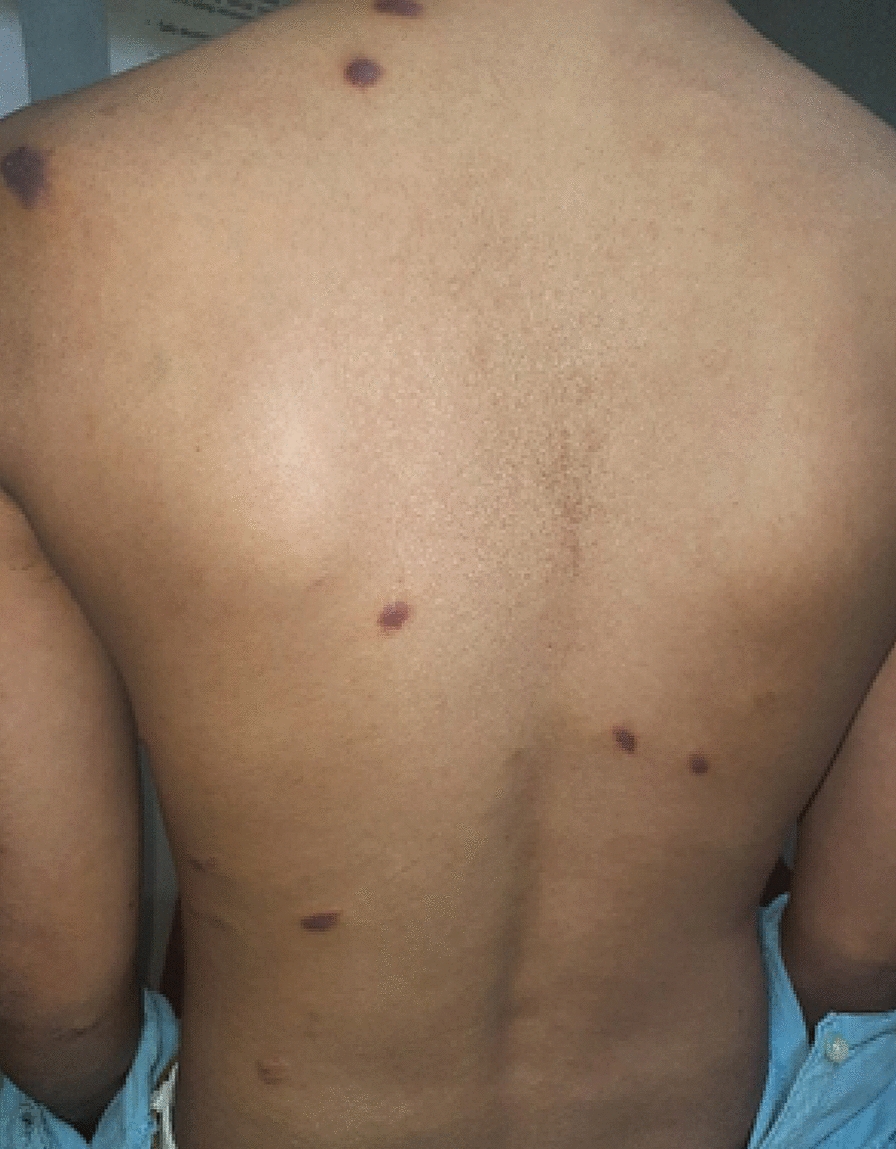
Purple, reddish-brown patches on the back side of the body

**Fig. 3:**
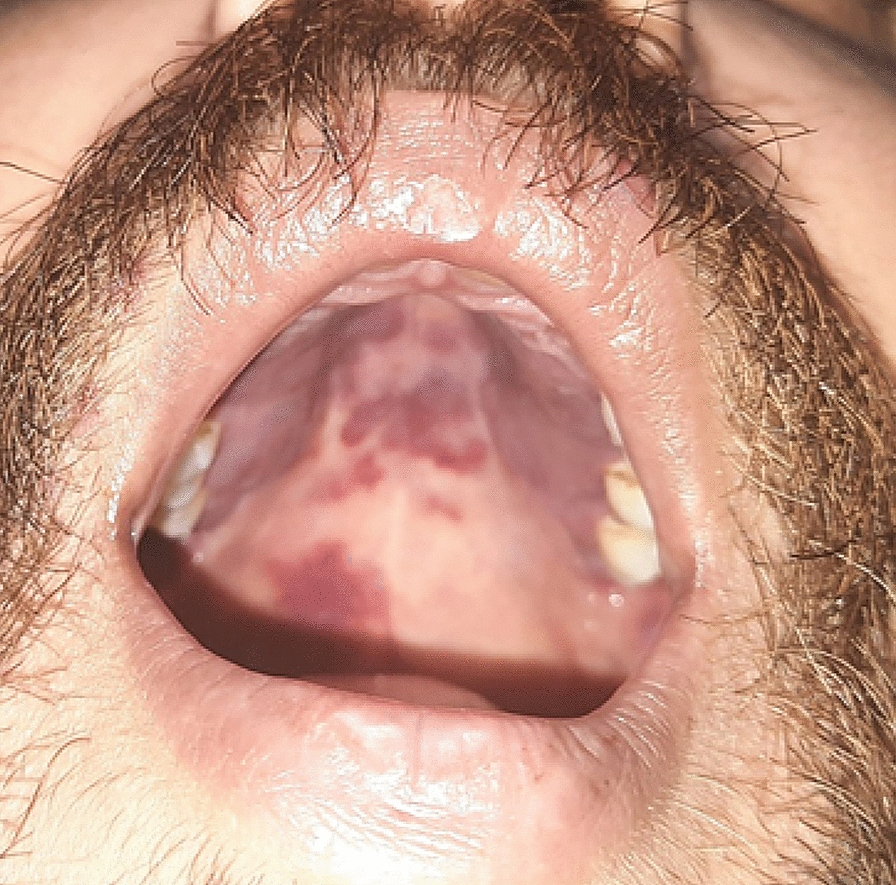
Patches inside the oropharynx

**Fig. 4:**
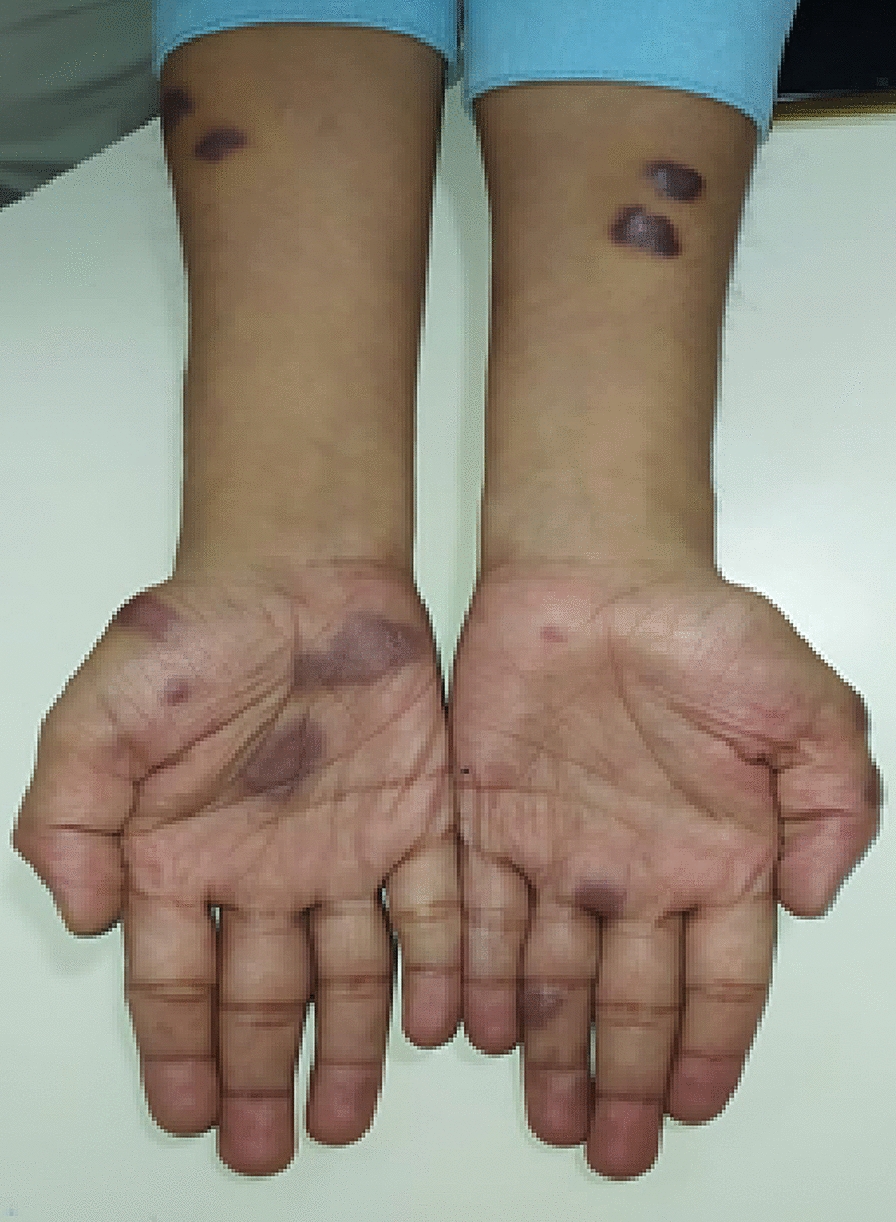
Well-defined, discrete, patches on forearm and palmer surface

**Fig. 5:**
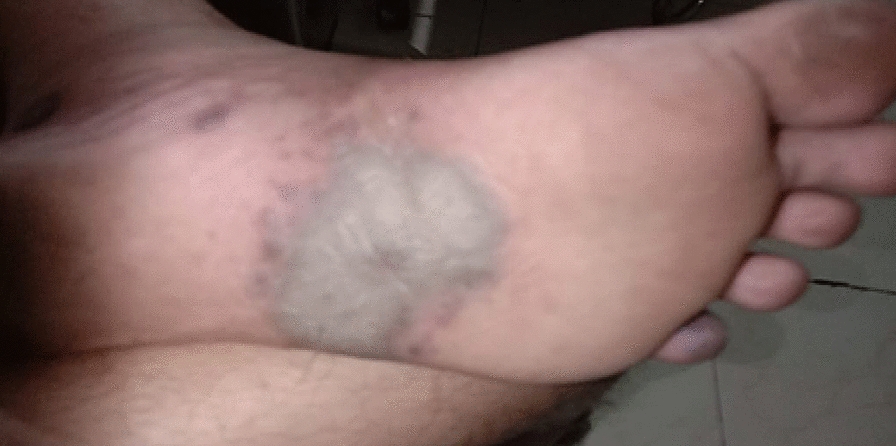
Well-defined, discrete patches on the plantar surface

We present a case of a 30-year-old unmarried male, engaged in same-sex sexual behaviors (MSM), who wanted to enroll in an ongoing oral Tenofovir Disoproxyl Fumarate containing PrEP demonstration project. He presented at the clinic with symptoms of purple, reddish-brown patches all over the body (Fig 1 and Fig 2) and mouth (Fig 3) for the past 3–4 months. The plaques were first noticed on the trunk 4 months back and gradual spread to other parts of the body happened over the next few months. There were no symptoms suggestive of respiratory distress or gastrointestinal involvement like bleeding or obstruction. Detailed history revealed that the patient was in a same-sex relationship since the age of 15 years and had multiple sexual contacts. He reported having unprotected sexual contact with multiple male partners during the last 3 months. He did not report any blood transfusion or intravenous use of illicit drugs for recreational purposes. The participant was afebrile (37.2 °C) on examination and the heart rate, respiratory rate, blood pressure, and BMI were 78 beats/minute, 22 breaths/minute, 100/70 mmHg, and 18.5 kg/m^2^ respectively. No neck lymphadenopathy or jugular engorgement was observed. Bilateral pitting pedal edema was noted during the general examination. No hepatomegaly or splenomegaly was observed on abdominal examination. The result of the neurological examination was normal. Dermatological examination showed the presence of multiple, well-defined, discrete, scaly, non-tender, non-blanching, non-itchy violaceous, raised macules and patches on the face, trunk, and extremities [palmer (Fig 4), planter (Fig 5)] and oropharyngeal mucosa with size varying between 3 mm and 4 cm in diameter with the classical truncal distribution. Plaques on the trunk were showing a typical ‘Christmas tree pattern’.

Blood and urine samples were collected for investigations. Enzyme-linked immunosorbent assay (ELISA) for HIV-1 was positive, and Rapid Plasma Reagin (RPR) test titer was 1:32. The urine sample was positive for *Chlamydia trachomatis on* Cartridge Based Nucleic Acid Amplification Test*.* The CD4 count was 226 cells per cubic millimeter of blood. Hepatitis B surface antigen and anti**-**hepatitis C virus antibody tests were negative. Ultrasonography of the chest revealed bilateral mild pleural effusion with a maximum thickness of 1 cm on either side. High-Resolution Computed Tomography (HRCT) revealed pleural effusion with bilateral infiltrates and was suggestive of infective etiology, most likely pulmonary tuberculosis. Cartridge-based Nucleic Acid Amplification (CB-NAAT) of sputum sample confirmed the infection as *Mycobacterium tuberculosis*. CSF examination showed proteins 42 mg/dl, Glucose 59 mg/dl, Total nucleated cells (predominantly lymphocytes) = 4. MRI brain showed minimal mucosal thickening in bilateral maxillary sinuses and ethmoid sinus. Baseline Haemogram and serum Biochemistry were unremarkable except for low hemoglobin levels. The serum sample was processed for Human Herpesvirus 8 (HHV-8) DNA PCR, which was found to be negative. To confirm the diagnosis of KS, a punch biopsy of the dermal lesion was taken. The section showed a dermis with multiple, irregular, jagged vascular channels extending to the subcutaneous tissue. These spaces partly surrounded pre-existing blood vessels and sweat glands at spaces. In addition, the dermis had fibro-collagenous tissue and inflammatory infiltrates composed of lymphocytes and plasma cells. A focal collection of spindle-shaped cells was also seen. The overlying epidermis showed hyperkeratosis and irregular hyperplasia. The histopathological findings confirmed the diagnosis of Kaposi Sarcoma. The patient was started on Anti-tubercular Treatment (ATT) for pulmonary tuberculosis. Highly Active Antiretroviral Therapy (HAART) was initiated after 2 weeks of initiation of ATT. After 1 month of ATT initiation, the patient developed an ATT-induced liver injury. Complete Blood Count (CBC) and Biochemistry done after 1 month of ATT initiation showed Haemoglobin = 10.6 gm/dl, Total Leucocyte Count (TLC) = 10.3 × 10^3^/μL, Platelet count: 115 × 10^3^/μL, Erythrocyte Sedimentation Rate (ESR) = 40 mm/hour, Mean Corpuscular Haemoglobin (MCH) = 32 pg, Mean Corpuscular Haemoglobin Concentration (MCHC) = 33.1 gm/dl, Mean Corpuscular Volume (MCV) = 97 fl, Mean Platelet Volume (MPV) = 11 fl, Total Bilirubin = 12.70 mg/dl, Direct bilirubin = 9.10 mg/dl, serum glutamic-oxaloacetic transaminase (SGOT) = 142 IU/L, serum glutamic pyruvic transaminase (SGPT) = 152 IU/L, Alkaline phosphatase (ALP) = 64 IU/l, serum creatinine = 0.67 mg/dl, serum uric acid = 16.6 mg/dl, serum urea = 15 mg/dl). Hence, he was put on liver sparing Anti Tubercular Therapy (ATT). The patient was then referred to a higher level of care for further management. He was found to have severe hypoalbuminemia leading to anasarca. During the hospital stay, he developed progressive breathlessness along with a worsening pleural effusion (left side). He was managed with repeated therapeutic thoracocentesis because of reaccumulating fluid. Cyto-morphology of the pleural fluid revealed features of primary effusion lymphoma. Flow cytometry of the pleural fluid was suggestive of PEL (large atypical cells expressing CD38, CD 56, and CD4, negative for pan B cells and T cell markers). CD 34 was negative. Video-assisted thoracoscopic surgery (VATS) could not be done due to the poor general condition of the patient. Considering the visceral involvement of the disease, systemic chemotherapy was initiated along with HAART and ATT. The prognosis of the case is poor and the same was informed to the patient in the language he understands.

## Discussion

KS is the most commonly seen malignancy among HIV-infected individuals. It typically starts with cutaneous involvement progressing to mucosal involvement and finally metastasizing to other visceral organs. With the introduction of antiretroviral therapy, the prevalence of KS is decreasing among HIV-infected patients however in a high-risk population, the incidence remains unchanged. The prognosis of KS often depends upon the stage of disease at the time of diagnosis. The attending physician should be very alert and watchful for the clinical manifestations of KS, especially in high-risk patients. It is important to have a high index of suspicion of KS and take detailed sociodemographic, sexual behavior, and clinical history for early diagnosis and initiation of immediate treatment.

In Pre HIV era, the incidence of KS was low. However, with the discovery of HIV, the incidence started increasing. Currently, the prevalence of KS in Asia and the USA is < 10% as compared to sub-Saharan Africa where the prevalence is > 90% [5﻿]. The recent literature reports an overall seroprevalence of 33% among MSM irrespective of their HIV status [6]. Chang Y CE et al. have reported the incidence of KS among HIV-infected people as 481.54 per 100,000 person-years [95% (CI) 342.36–677.32 per 100,000 person-years] while the incidence is highest among HIV-infected MSM (1397.11 per 100,000 person-years; [95% (CI)  870.55–2242.18 per 100,000 person-years] [1]. The incidence of KS is found to be significantly lower in females than in males (IRR 3.09; 95% CI 1.70–5.62) [6].

A total of 25 cases of Kaposi sarcoma have been reported in India between 1993 and 2019 (i.e. on an average of 1 case/year) [6]. Despite the number of cases of KS being low in India, cases are seen in LGBT (Lesbian, Gay, Bisexual, and Transgender) population in India [5]. LGBT group is known to experience social discrimination and are at high risk of HIV/AIDS and sexually transmitted diseases. Social stigma and discrimination experiences faced by them at health care facilities make healthcare utilization suboptimal leading to unreported or ill-reported cases most of the time. Considering the increasing occurrence of KS cases among this group, importance should be given on emphasizing the early detection of KSHV. The evidence is available for saliva and genital secretions as the mode of transmission of HHV-8 [7–9]. A qualitative study conducted among MSM revealed a lack of knowledge about voluntary HIV testing and counseling centers and low levels of susceptibility perception in them [10]. Despite the availability of ART and Test and Treat guidelines, delayed HIV diagnosis is common. This leads to delayed presentation to the health care system. Also in India, the majority of suspected cases are not further investigated for detection of HHV-8 and biopsy of the lesions due to resource limitations that lead to non-confirmation of diagnosis. A major challenge in resource-poor settings is poor self-risk perception, delayed diagnosis, and access to treatment. PrEP is efficacious among MSM and recommended by CDC for use in high-risk MSM to prevent the risk of acquisition of HIV [11]. The correct and consistent use of PrEP might prevent the acquisition of HIV thereby delaying the development of the AIDS-defining illness stage rapidly. Considering the higher rates of infection among MSM there is also a need for research on a vaccine against HHV-8 to prevent KS among them.

## Data Availability

All supporting reports and photos are available with the corresponding author.

## References

[1] Brodt HRKB, Gute P, Knupp B, Staszewski S, Helm EB (1997). Changing incidence of AIDS-defining illnesses in the era of antiretroviral combination therapy. Aids.

[2] Gao SJKL, Li M, Zheng W, Parravicini C, Ziegler J, Newton R, Rinaldo CR, Saah A, Phair J, Detels R, Chang Y, Moore PS (1996). KSHV antibodies among Americans, Italians, and Ugandans with and without Kaposi's sarcoma. Nat Med.

[3] Franceschi SGM (1995). Epidemiology of classic Kaposi's sarcoma, with special reference to Mediterranean population. Tumori.

[4] Renwick NHT, Weverling GJ, Dukers NH, Simpson GR, Coutinho RA, Lange JM, Schulz TF, Goudsmit J (1998). Seroconversion for human herpesvirus 8 during HIV infection is highly predictive of Kaposi's sarcoma. Aids.

[5] Liu ZFQ, Zuo J, Minhas V, Wood C, Zhang T (2018). The worldwide incidence of Kaposi’s sarcoma in the HIV/AIDS era. HIV Med.

[6] Das PCN, Chatterjee K, Choudhuri T (2020). Kaposi’s sarcoma-associated herpesvirus related malignancy in India, a rare but emerging member to be considered. Virus Dis.

[7] Pauk JHM, Brodie SJ, Wald A, Koelle DM, Schacker T, Celum C, Selke S, Corey L (2000). Mucosal shedding of human herpesvirus 8 in men. N Engl J Med.

[8] Casper CRM, Huang ML, Pauk J, Lampinen TM, Hawes SE, Critchlow CW, Morrow RA, Corey L, Kiviat N, Wald A (2004). HIV infection and human herpesvirus-8 oral shedding among men who have sex with men. JAIDS J Acquir Immune Defic Syndr.

[9] Casper CKE, Selke S, Kuntz SR, Wang J, Huang ML, Pauk JS, Corey L, Wald A (2007). Frequent and asymptomatic oropharyngeal shedding of human herpesvirus 8 among immunocompetent men. J Infect Dis.

[10] Bangar UMS, Kumar S, Mahapatra A, Singh S, Kohli R, Verma A, Verma V, Saravanamurthy P, George B, Rajan S, Sahay S, Health care access and programmatic gaps: facilitators and barriers for men having sex with men (MSM) in rural India, E-Poster number: PEC0671, Category C-39, presented in the 23rd International AIDS Conference, 6-10 July 2020, Virtual AIDS 2020. https://www.aids2020.org/wp-content/uploads/2020/09/AIDS2020_Abstracts.pdf. Accessed 6 July 2022.

[11] Centers for Disease Control and Prevention: US Public Health Service: Preexposure prophylaxis for the prevention of HIV infection in the United States—2021 Update: a clinical practice guideline. 2021. https://www.cdc.gov/hiv/pdf/risk/prep/cdc-hiv-prep-guidelines-2021.pdf.

